# Supplementary material for: An integrated human forebrain organoid reveals microglia-mediated CD8+ T cell recruitment and neuroimmune dysfunction in Alzheimer’s disease pathology
Source: eLife. 2026 Oct 20;15:RP111730. doi: 10.7554/eLife.111730 (PMC13682712; doi:10.7554/eLife.111730)
Supplement: Peer review history

# Editor report: eLife Assessment

**DOI:** 10.7554/eLife.111730.3.sa0

**Authors:** Florent Ginhoux

**Affiliations:** Singapore Immunology Network Singapore

**Keywords:** Convincing, Valuable

This study presents a **valuable** human organoid platform for investigating neuron–immune interactions in Alzheimer's disease and modeling the interplay between innate and adaptive immunity in the context of amyloid pathology. The system has the potential to advance the field by enabling the study of human-specific neuroimmune interactions that is difficult to recapitulate in rodent models. The evidence on the microglia–T cell crosstalk is **convincing**. The study will interest researchers in neuroimmunology and Alzheimer's disease.

# Referee report: Reviewer #2 (Public review)

**DOI:** 10.7554/eLife.111730.3.sa1

Summary:

In this study, the authors developed a human forebrain organoid model that incorporates both iPSC-derived microglia and CD8⁺ T cells, allowing them to recreate and investigate multicellular aspects of AD pathology in a human-relevant system.

Their findings show that microglia help clear amyloid-β deposits, but they also promote inflammatory responses. Activated microglia recruit CD8⁺ T cells by releasing the chemokines CCL4, CCL5, and CXCL10, which signal through the receptors CCR1/CCR5 and CXCR3. Pharmacological inhibition of CCR5 or CXCR3 prevents T-cell recruitment and alters autophagy pathways in a microglia-dependent manner.

Strengths:

The manuscript has several strengths, including its innovative multicellular organoid model, the combination of complementary experimental approaches, and the identification of potentially relevant immune signaling pathways.

Comment on the revised version.

Overall, the revised manuscript is considerably improved, and the major conceptual concerns raised in the initial review have been adequately addressed.

# Author comment: Author response

**DOI:** 10.7554/eLife.111730.3.sa2

**Authors:** Yilin Feng, Feifei Yu, Jiyuan Tang, Honghui Zheng, Zitian Wang, Shaohua Ma

**Affiliations:** Tsinghua University Shenzhen China; Tsinghua University Shenzhen China; Tsinghua University Shenzhen China; Tsinghua University Shenzhen China; Tsinghua University Shenzhen China; Tsinghua University Shenzhen China

The following is the authors’ response to the original reviews.

**Public Reviews:**

**Reviewer #1 (Public review):**

Summary:

A growing body of evidence indicates that Alzheimer's disease is not simply a disease of neurons accumulating toxic protein aggregates, but one in which the immune system, both its resident brain component and its circulating peripheral arm, plays an active and sustained role. Understanding how these two immune compartments interact with one another and with diseased neural tissue has been hampered by the fact that the mouse immune system differs fundamentally from the human one in ways likely to matter for disease progression. The authors set out to address this gap by building a modular laboratory model that brings together three human cell types in a three-dimensional setting: brain organoids derived from human stem cells to provide a neural substrate, stem cell-derived brain immune cells (microglia) to represent the resident immune compartment, and circulating immune cells (CD8-positive T cells) harvested from human blood to represent the peripheral adaptive immune response. By exposing this tri-cellular system to a toxic form of amyloid protein, the hallmark aggregating molecule of Alzheimer's disease, the authors aimed to dissect, step by step, how microglia respond to amyloid stress, what inflammatory signals they release as a consequence, and whether those signals are sufficient to attract T cells into the neural environment. They further aimed to test whether blocking the molecular receptors that guide T cell movement could interrupt this process, with the broader goal of positioning the platform as a tool for human-relevant drug screening.

Strengths

The conceptual architecture of the platform is one of its clearest strengths. The decision to add immune components in a stepwise, modular fashion, first characterising the neural response to amyloid, then adding microglia, then adding T cells, makes it possible to attribute observed changes to specific cellular contributions in a way that a more complex all-at-once model would not allow. This staged design is well thought-through, and its logic is clearly communicated. The combination of single-cell transcriptional profiling, calcium imaging for real-time functional readouts, transwell migration assays, and protein secretion measurements gives the study a genuinely multi-modal character that goes beyond what purely transcriptomic or purely imaging-based approaches can offer. The observation that T cells failed to migrate toward amyloid-treated organoids in the absence of microglia is a clean and conceptually important result, clearly supporting the idea that the resident immune response acts as an intermediary between amyloid pathology and the recruitment of peripheral immune cells. The identification of specific chemokine receptor pathways mediating T cell movement and the demonstration that pharmacological blockade of those receptors reduces migration and provide a degree of mechanistic resolution useful for thinking about future therapeutic strategies.

Weaknesses

Despite these strengths, several aspects of the work as presented substantially limit the confidence one can place in its conclusions.

The most consequential issue concerns the origin of the cells used in the model. The three cellular components: the brain organoids, the microglia, and the T cells are derived from genetically unrelated individuals. The T cells, in particular, come from healthy blood donors unrelated to the stem cell lines used to generate the neural tissue. This means the immune cells and the tissue they are interacting with carry different molecular identity markers (the proteins that the immune system uses to distinguish self from non-self). In this setting, any T cell activation or directed movement could reflect a generic rejection-like response to foreign tissue rather than a disease-relevant, chemokine-directed recruitment process. This is not a subtle concern: it represents a fundamental ambiguity at the heart of the model's central finding, and it is not acknowledged anywhere in the manuscript. For the transwell migration data to be interpretable as a model of Alzheimer's disease rather than of immune incompatibility, the authors would need to demonstrate that migration is driven by the specific chemokine environment and not by the genetic mismatch between cells, for example, using cells from the same donor or from matched donors, or by showing that blocking identity-marker recognition does not alter migration.

A related concern is that the T cells used are from healthy individuals, whereas T cells from people with Alzheimer's disease are known to differ in their activation state, surface receptor expression, and functional behaviour. The platform cannot yet claim to model the specific T cell biology of Alzheimer's disease until disease-relevant T cells are incorporated.

Beyond this foundational issue, the study frequently describes findings in causal terms that the experimental design does not support. The resident immune cells are said to "drive" T cell recruitment and "establish" a feedback loop. These are strong mechanistic claims. The evidence presented indicates that when microglia are present, more T cells migrate, and that blocking T cells receptors reduces migration. What is missing is direct evidence that the specific molecules measured, particularly the chemokines CCL4 and CCL5, are the agents responsible, as opposed to other signals also present in the conditioned environment. No experiment directly neutralises these chemokines to test whether their removal is sufficient to abolish T cell recruitment. Without such an experiment, the receptor-blocking data show only that the receptors matter, not that the measured ligands are the ones activating those receptors.

The abstract describes one particular molecule, CXCL10, as a contributor to T cell recruitment, but the data in the paper itself show no significant change in CXCL10 levels between conditions. This discrepancy between the abstract and the results is misleading to readers who may not read the figures in detail.

The single-cell sequencing data, which form the basis for claims about changes in cell populations following amyloid treatment or microglia addition, are presented without validation of the cell type labels against established reference datasets from human brain tissue. The proportional shifts in cell populations between conditions (Figures 1H and 3E) are described as significant findings but are shown without any statistical test appropriate for this type of compositional data. Comparisons of cell-type proportions derived from single-cell sequencing require specialised statistical approaches that account for the interdependence of proportions and the variability between samples; standard tests are not appropriate here, and none are applied.

There is also an unresolved inconsistency in the age at which the organoids were analysed by single-cell sequencing: the text states day 90, while the figure legend states day 60, and the methods section contains a passage describing experimental conditions (including a cholesterol treatment and a drug called semaglutide) that are entirely unrelated to this study and appear to have been copied from a different manuscript. These issues raise concerns about the rigour of the manuscript preparation and should be corrected.

Finally, the sample sizes underpinning several key conclusions are small (typically three to four organoids per group), particularly for the protein-secretion measurements used to identify the inflammatory signals responsible for T cell recruitment. While organoid studies are inherently limited in scale, the strength of the mechanistic claims made here would benefit from larger sample size or independent experimental replication.

Conclusion:

The authors have built a platform that is conceptually well-conceived and generates data consistent with a role for microglia in bridging amyloid pathology and T cell recruitment. In that sense, they have made meaningful progress toward their stated aims. However, the platform, as described, cannot yet deliver the human-specific mechanistic insight it claims to provide, primarily because the non-autologous configuration of the model introduces an uncontrolled variable that confounds the interpretation of the immune interaction data. The claim to have provided "the first human-specific mechanistic demonstration" of microglial activation as a bridge between amyloid pathology and adaptive immune recruitment is not supported by the evidence presented. The data are consistent with this interpretation but do not establish it.

The general approach, building increasingly complex human neural-immune models by adding components in a controlled, stepwise manner, is a valuable direction for the field and one that other groups working on neuroinflammation will find useful to consider. The combination of live calcium imaging and transcriptional profiling in the same experimental system is a practical contribution that demonstrates the kind of multi-modal readout this class of model can support. If the autologous confound is resolved in future iterations and if the mechanistic claims are grounded in more direct experimental evidence, this type of platform could become a genuinely useful tool for investigating human neuroimmune biology and for screening candidate therapeutic compounds in a human-relevant context. As currently presented, however, readers and researchers considering adopting this approach should be aware that the immune interaction data may reflect genetic mismatches between cell sources rather than disease-specific biology, and that the causal conclusions drawn from the chemokine and migration data go beyond what the experiments can support.

**Reviewer #2 (Public review):**

Summary:

In this study, the authors developed a human forebrain organoid model incorporating both iPSC-derived microglia and CD8^+^ T cells, enabling them to recreate and investigate multicellular aspects of AD pathology in a human-relevant system.

Their findings show that microglia help clear amyloid-β deposits, but they also promote inflammatory responses. Activated microglia recruit CD8^+^ T cells by releasing the chemokines CCL4, CCL5, and CXCL10, which signal through the receptors CCR1/CCR5 and CXCR3. Pharmacological inhibition of CCR5 or CXCR3 prevents T-cell recruitment and alters autophagy pathways in a microglia-dependent manner.

Strengths:

The study presents a versatile human organoid platform for investigating neuron-immune interactions in Alzheimer's disease. It highlights the critical role of microglia-driven recruitment of CD8^+^ T cells in sustaining neuroinflammation and identifies CCR5 and CXCR3 signaling pathways as promising therapeutic targets for neuroinflammatory conditions.

This study is interesting and presents novel findings supported by state-of-the-art approaches, including single-cell RNA sequencing, a three-dimensional cerebral organoid model, and co-culture systems involving two distinct immune cell populations.

Weaknesses:

Several aspects of the study require clarification and further improvement. For example:

(1) Figure 1H is missing statistical analyses.

(2) The scRNA-seq analysis shows a reduction in the proportion of cells occupying transcriptional states associated with later pseudotime values, which the authors interpret as evidence that Aβ treatment inhibits neuronal maturation. However, the data presented do not appear sufficient to support this conclusion. An alternative explanation is that Aβ preferentially affects the survival of more mature neuronal populations, leading to their depletion, consequently, an apparent enrichment of cells at earlier pseudotime states. Therefore, the observed pseudotime shift does not necessarily demonstrate impaired maturation per se. The authors should revise the interpretation of these results in the first paragraph and either provide additional evidence supporting a maturation defect or discuss alternative explanations such as selective loss of mature neurons.

(3) A similar concern applies to the scRNA-seq data presented in Figure 3. The authors interpret the shift toward later pseudotime states in the presence of microglia as evidence of enhanced neuronal maturation. However, the data do not exclude alternative explanations. For instance, microglia may preferentially promote the survival of more mature neuronal populations or protect them from cell death, thereby increasing their relative abundance in the dataset. Consequently, the observed pseudotime distribution cannot be taken as direct evidence of enhanced maturation. The authors should revise their interpretation accordingly and discuss the possibility that the observed effect reflects differential survival rather than accelerated neuronal maturation.

(4) In Figures 4A-E, the authors should report the levels of the secreted proteins in pg/mL instead of relative values, as this would better reflect the actual amounts produced. In Figure 4H, the inhibitor-treated control T-cell samples should be included. Furthermore, it should be explicitly stated that the inhibitor-treated data points currently shown refer to T cells cultured in the presence of myeloid Aβ.

**Recommendations for the authors:**

**Reviewer #1 (Recommendations for the authors):**

(1) Addressing the allogeneic confound

To determine whether observed T cell migration reflects chemokine-driven recruitment rather than allogeneic recognition, the authors should perform one or more of the following: (a) repeat transwell migration assays using HLA-matched donors for T cells and iPSC lines; (b) include a blocking condition using anti-HLA class I antibody to suppress allogeneic recognition and test whether migration is reduced; (c) compare migration toward conditioned media alone (without cells) versus co-culture conditions to establish whether secreted factors are sufficient to drive migration independently of direct cell contact. If HLA-matched material is not currently available, the authors could, at a minimum, use conditioned media transfer experiments to dissociate chemokine-mediated from contact-mediated effects.

We agree this is an important limitation. We have not performed HLA-matched migration assays, anti-HLA class I blockade, or conditioned-media-only migration experiments. We note that in our transwell assay design (Fig. 4G–H), T cells are seeded in the upper chamber and organoids in the lower chamber, separated by a porous membrane; while this precludes direct T cell–organoid cell contact, live organoid/microglial cells are present throughout the assay rather than their conditioned medium alone, so we have not directly tested part (c) as the reviewer describes it.

We would also note that the CD8^+^ T cells used in this assay were derived from a single healthy donor, while the organoids and iMGLs were derived from a separate iPSC line (DYR0100); the same T cell donor and the same organoid/microglial line were used across all conditions (CTR, Aβ42, Aβ42+iMGL). The T cell–organoid pairing is therefore allogeneic, but identical in every condition. Any contribution of allogeneic recognition to baseline migration should, in principle, be present equally across conditions and would not by itself account for the increased migration specifically toward the Aβ42 and Aβ42+iMGL conditions relative to CTR.

(2) Direct validation of CCL5 as the responsible ligand

To substantiate the claim that microglia-derived CCL5 is responsible for T cell recruitment, a CCL5-neutralising antibody should be added to the transwell assay. Similarly, a CCL4-neutralising antibody should be tested independently. This would allow the authors to attribute the migration effect to specific molecules rather than to the overall conditioned environment and would substantially strengthen the mechanistic interpretation.

We appreciate this comment and agree that ligand-neutralization experiments would substantially strengthen the mechanistic interpretation. We have not performed these experiments in the current study but will consider including CCL5- and CCL4-neutralizing antibody conditions in future follow-up work. We have revised the Results/Discussion to make clear that our receptor-blockade data (Fig. 4H) establish that CCR5/CXCR3/CCR1 signaling on T cells is necessary for recruitment, but do not by themselves attribute this effect to CCL4 or CCL5 specifically, as opposed to other chemokines present in the conditioned environment.

(3) Cell-type annotation validation for single-cell data

The authors should add a supplementary figure showing a dot plot or heatmap of canonical marker gene expression across all annotated clusters, cross-referenced to at least one published human brain atlas or human organoid single-cell reference dataset. Module score analysis using published cell-type gene signatures (e.g., Velmeshev et al. 2019, for neuronal subtypes or established microglial signature sets) would further validate the annotations. The specific software tool and reference dataset used for annotation should be explicitly named in the Methods section.

We thank the reviewer for this suggestion. Canonical marker gene expression supporting our cluster annotations is provided in Supplementary Figure S1 (violin plots of marker genes across annotated clusters).

(4) Statistical testing for cell-type proportion comparisons

Figures 1H and 3E should be reanalysed using a compositional statistical framework. The authors are encouraged to use the propeller method (Phipson et al., Bioinformatics, 2022) or scCODA (Büttner et al., Nature Communications, 2021), both of which are designed for this type of comparison in single-cell data. Individual organoid-level proportion values should be shown as overlaid data points to make biological variability is visible.

We agree and have reanalysed both comparisons with scCODA (Büttner et al., 2021), using cell-type counts aggregated per library (n = 2 per condition). Because scCODA estimates are conditional on a reference population, we specified migrating neurons as reference — the only population with a near-unity fold change (1.19×) and no credible change under any alternative reference. The results are shown in Supplementary Table 1.

We note that scCODA reports posterior inclusion probabilities rather than P values, and have removed P-value notation from all proportion comparisons. Figures 1H and 3E now display composition for each individual library as separate stacked bars, so that between-replicate variability is directly visible.

(5) Functional validation of microglial-promoted neuronal maturation

To support the claim that microglia promote neuronal maturation, at least one functional or morphological measurement attributable specifically to the microglia co-culture condition should be provided. Suggested approaches include: quantification of neurite length or branching complexity by automated image analysis comparing organoid slices with and without microglia; or synaptic puncta density measurements (already used elsewhere in the manuscript) comparing Aβ42 and Aβ42+iMGL conditions.

Thanks a lot for pointing this out. We agree that trajectory inference alone cannot support a claim that microglia promote neuronal maturation, and we regret that we are unable to provide the requested measurement: synaptic puncta quantification in this study was performed only for the CTR and Aβ42 conditions, and the material required to extend it to Aβ42+iMGL is no longer available. We have therefore revised the interpretation rather than defend the original claim.

We now describe the effect as a shift in organoid cell-type composition and transcriptional state toward more differentiated populations, rather than as microglia-driven neuronal maturation. The Results text has been changed from "…indicating that microglia promote neuronal maturation and partially reverse the Aβ42-induced immature state" to:

" Pseudotime analysis showed that microglial co-culture shifted cells toward higher pseudotime values compared with Aβ42 alone (Fig. 3F–G), consistent with a shift in organoid cell-type composition toward more differentiated populations (Fig. 3E) and a partial reversal of the Aβ42-associated skew toward progenitor states.”

(6) Chemokine secretion follow-up after T cell addition

To begin closing the proposed feedback loop, the authors should measure CCL4 and CCL5 levels (and ideally a broader cytokine panel) in conditioned media from the Aβ42+iMGL+T condition and compare them with those from Aβ42+iMGL. If T cells amplify microglial activation (as shown by calcium imaging), one would predict increased chemokine output. Including this measurement would substantially strengthen the feedback loop claim.

We would first clarify the scope of our claim. The feedback loop described in the manuscript operates at the level of cellular activation state: microglia-derived chemokines recruit CD8^+^ T cells (Fig. 4A–E, 4G–H), and recruited CD8^+^ T cells in turn amplify microglial activation (Fig. 4L). Each step is supported by direct measurement, and we do not claim that T cells increase microglial chemokine output or drive a further round of recruitment.

We agree that measuring chemokine secretion after T cell addition would test an important extension of this model — whether T cell-driven amplification of microglial activity translates into increased chemokine output and thus a self-sustaining recruitment cycle. We are unable to perform this experiment within the revision period, as the tri-culture conditioned media were not retained.

We would also note that this measurement is not straightforward in bulk conditioned media. CD8^+^ T cells are themselves major producers of CCL4 and CCL5, releasing preformed CCL5 from cytotoxic granules within hours of activation, so an increase in the Aβ42+iMGL+T condition could not be attributed to microglia. A rigorous test would require cell-type-resolved output — intracellular cytokine staining, single-cell profiling of the tri-culture, or physical separation of the two populations — and we now identify this explicitly as the next step.

(7) Time-course data for the protective-to-pathological transition

The dual functionality narrative requires temporal data to be credible. The authors should consider measuring Aβ clearance, pseudotime distribution, and chemokine secretion at multiple time points after microglial addition (e.g., 6, 24, and 48 hours) to determine whether the protective and inflammatory outputs are sequential or whether they co-occur from the outset.

We agree that a single 48-hour timepoint cannot determine whether protective and inflammatory microglial outputs are sequential or concurrent, and we are unable to add a time course within the revision period.

We have therefore moderated our interpretation. The Discussion previously stated that "microglial activation is not a binary state but a spectrum, where sustained Aβ stress drives the transition from a protective to a pathological, T-cell-recruiting state." This has been revised to: "This duality indicates that microglial activation is not a binary state: within the Aβ42 environment, clearance and neuroprotective functions are detectable alongside inflammatory, T-cell-recruiting outputs at the same timepoint." The temporal claim implied by "transition" has been removed, as our data establish co-occurrence at 48 hours rather than a sequence.

Recommendations for writing and presentation

(8) Abstract correction

CXCL10 must be removed from the mechanistic description of T cell recruitment in the abstract, as Figure 4E shows no significant change in CXCL10. The abstract should also moderate the language around "establishing a feedback loop" to reflect that the loop was observed and partially characterised rather than mechanistically closed.

CXCL10 has been removed from the mechanistic description in the Abstract, as Fig. 4E shows no significant change in its secretion. CXCR3 has been retained, as pharmacological blockade reduced T cell migration (Fig. 4H), but is now presented as a receptor-level finding without an identified ligand. We have also moderated the concluding clause, which now describes a neuroimmune circuit in which recruited T cells amplify microglial activation, rather than stating that a feedback loop is established. The Abstract sentence now reads: Using this platform, we demonstrate that microglia mediate amyloid-β clearance and shift organoid cell composition toward more differentiated states, but also become inflammatory and recruit CD8^+^ T cells through CCL4/CCL5 signaling via CCR1, CCR5, and CXCR3, forming a neuroimmune circuit in which recruited T cells further amplify microglial activation.

(9) Resolve the day 90 / day 60 inconsistency

The discrepancy between "day 90" in the Results text (line 88) and "day 60" in the Figure 1G legend must be corrected throughout the manuscript.

"Day 90" in the Results text (line 88) has been corrected with “day 60”.

(10) Rewrite the scRNA-seq library preparation methods

The methods section (lines 357-360) must be rewritten to describe the actual experimental conditions used in this study. The passage referring to cholesterol-treated and semaglutide-treated organoids on day 30 must be removed entirely.

The methods section has been now revised as: For isolation of iPSC-derived forebrain cells, cells were collected from CTR and Aβ42-treated organoids at day 60, and from Aβ42-treated organoids with and without a 48-hour iMGL co-culture (Aβ42 and Aβ42+iMGL). Two biological replicates were included in each group. Three organoids were pooled within each replicate and washed three times with pre-chilled PBS. Single-cell suspensions were prepared by incubating with 0.5 mg/mL papain for 30 min at 37 °C in a water bath. Suspensions were filtered through a 70 µm cell strainer (BD Falcon, 352350). After centrifugation at 500 g for 5 min, the cell pellet was resuspended in PBS + 0.01% BSA.

(11) Report the number of organoids per scRNA-seq replicate

The Methods section should explicitly state how many organoids were pooled per biological replicate for each single-cell sequencing experiment and how many replicates were included per condition.

The Methods section has been revised to state explicitly the number of organoids pooled per biological replicate and the number of replicates per condition for each single-cell experiment, as described in our response to comment (10). Two biological replicates were included per condition, each prepared from 3 pooled organoids.

(12) Attribute iPSC lines to specific experiments

A table or supplementary note should specify which cell line (H9, DYR0100, or DXR0109B) was used for each experiment and figure. If different lines were used interchangeably within the same figure, this should be stated and any resulting variability discussed.

All organoid data presented in this manuscript were generated from a single line, DYR0100; the H9 and DXR0109B lines were not used for any experiment reported here. We have removed them from the Methods to avoid ambiguity, and the Methods now state that DYR0100 was used for all organoid experiments. No experiment or figure combines material from different lines.

(13) Moderate the use of "human-specific"

Throughout the manuscript, claims of human specificity should be restricted to contexts where a direct comparison with rodent data has been performed or cited. When the platform uses only human cells, the appropriate phrasing is "human-derived" or "in a human cellular context" rather than "human-specific mechanisms."

We have replaced "human-specific" throughout with "human-derived" or "in a human cellular context," at four locations: the Abstract, the final paragraph of the Introduction, the second paragraph of the Discussion, and the legend of Fig. 4O.

(14) Revise the "first demonstration" claim

The claim to provide the "first human-specific mechanistic demonstration" (line 218) is not substantiated and should be removed or substantially qualified.

This claim has been revised.

(15) Immunofluorescence panel legibility

Protein label text in Figures 1B-E and 3B should be rendered in a contrasting colour (e.g., white with a black outline, or placed on a coloured background tab) so that they are readable against the fluorescence image background. Scale bars should be present and consistent across all panels within a figure.

Protein label text in Figures 1B–E and 3B has been re-rendered in a contrasting colour so that it is legible against the fluorescence background. Scale bars are present in all panels, and their dimensions are stated in the corresponding figure legends.

(16) Remove or justify the Animals section in Methods

If no mouse data are presented in the paper, the Animals section should be removed. If mouse experiments were performed and the data are available, a corresponding figure or supplementary figure should be included.

Animals section description has been removed from Methods.

Corrections & Clarifications

(17) The following must be corrected before resubmission:

(a) Replace "(ref)" placeholders on lines 46 and 63 with full citations.

(b) Replace "(cell reports 2023)" on line 48 with a properly formatted citation. This appears to refer to Feng et al., Cell Reports 42, 113313 (2023), which is already reference 9 in the list - it should simply be cited as such.

(c) Reference 14 (Feng et al., STAR Protoc, glioma organoid protocol) does not support the CCL5-CCR5 claim for which it is cited and should be replaced with an appropriate reference.

(d) Reference 20, cited in the text (line 56), does not exist in the reference list. The intended citation must be identified and added.

(e) Reference 3 (Michell-Robinson et al.) is listed but not cited in the text. It should either be cited where appropriate or removed from the list.

We have carefully reviewed the manuscript and corrected the incomplete and incorrect references.

(18) GEO accession numbers

The manuscript states that bulk RNA-seq data have been deposited at GEO but does not provide an accession number. The accession number must be included in the Data Availability section.

Accession numbers have been added to the Data Availability section: bulk RNA-seq data are deposited under GSE339404 and single-cell RNA-seq data under GSE339602. Both datasets are publicly accessible.

(19) Single-cell RNA-seq data availability

The single-cell RNA-seq data are not mentioned in the Data Availability statement. These data should also be deposited in a public repository (GEO or a single-cell-specific repository such as the Human Cell Atlas data portal), and the accession number should be provided.

Mentioned in comment (18).

(20) Zenodo DOI

The Code Availability section states that code has been deposited at Zenodo, but no DOI is provided. The Zenodo DOI must be included.

Zenodo DOI has been added in the Methods: Single-cell RNA-seq data have been deposited at GEO under accession GSE339602 and bulk RNA-seq data under accession GSE339404; both are publicly accessible as of the date of publication. Microscopy data are available at Zenodo (DOI: 10.5281/zenodo.21686712). All other raw data, including cytokine array images, and all original code are available at https://github.com/FengYilinElaine/2-3D-AD-oranoids.

(21) Ethical approval for human blood collection

The manuscript states that blood was collected from healthy donors at Zhangzhou People's Hospital but does not mention institutional ethical approval for this collection or informed consent procedures. A statement confirming ethical approval (with approval number) and donor-informed consent must be added to the Methods section in accordance with eLife's policies on human subjects research.

Ethical approval for this study was granted by the First Affiliated Hospital of Zhengzhou University (2021-KY-0875-003) and Tsinghua Shenzhen International Graduate School (2024-F125). It has been added to the Methods.

(22) Microscopy data availability

The manuscript states that microscopy data will be shared upon request. eLife's data policy requires that primary data supporting the figures be made publicly available at the time of publication. The authors should deposit representative raw imaging data (or processed calcium imaging traces) in an appropriate repository such as Figshare, Zenodo, or the Image Data Resource and provide the link in the Data Availability section.

Mentioned in comment (20)

**Reviewer #2 (Recommendations for the authors):**

This study is interesting and presents novel findings supported by state-of-the-art approaches, including single-cell RNA sequencing, a three-dimensional cerebral organoid model, and co-culture systems involving two distinct immune cell populations.

These innovative methodologies provide valuable insights into the mechanisms under investigation. Nevertheless, several aspects of the study require clarification and further improvement.

The authors should address the points in the public review and the following additional points:

(1) Throughout the manuscript, the figure legends contain conclusions and interpretations of the data. These statements should be removed from the figure legends and incorporated into the Results section, while the legends should be restricted to describing the experimental conditions and the data displayed.

Revised manuscript and legends fixed the problem.

(2) The reference list and in-text citations appear to be incomplete. In several instances, the manuscript contains placeholder citations (e.g., "Ref") instead of full references. The authors should carefully review and complete all citations.

We have carefully reviewed the manuscript and corrected the incomplete and incorrect references. All placeholder citations have been replaced with the appropriate references, and the reference list and in-text citations have been checked for completeness.
